# My Train Talks to Me: Participatory Design of a Mobile App for Travellers with Visual Impairments

**DOI:** 10.1007/978-3-030-58796-3_2

**Published:** 2020-08-10

**Authors:** Beat Vollenwyder, Esther Buchmüller, Christian Trachsel, Klaus Opwis, Florian Brühlmann

**Affiliations:** 8 Institute Integriert Studieren, JKU Linz, Linz, Austria; 9 Jack Baskin School of Engineering, UC Santa Cruz, Santa Cruz, CA USA; 10 Dipartimento di Meccanica, Politecnico di Milano, Milan, Italy; 11 Support Centre for Students with Special Needs, Masaryk University Brno, Brno, Czech Republic; 12 Center for Cognitive Psychology and Methodology, Department of Psychology, University of Basel, Basel, Switzerland; 13 Swiss Federal Railways, Bern, Switzerland

**Keywords:** User experience, Digital accessibility, Participatory design, People with visual impairments, Case study

## Abstract

Travellers with visual impairments may face substantial information gaps on their journeys by public transport. For instance, information displayed in trains, as well as on departure boards in train stations and on platforms, are often not available in acoustic or tactile form. Digital technologies, such as smartphones or smartwatches, can provide an alternative means of access. However, these alternatives do not guarantee that the user experience is comparable in value, quality and efficiency. The present case study details a participatory design process, where travellers with visual impairments co-designed a mobile app. The goal was to tackle information gaps on journeys by public transport and to learn how participatory design can facilitate the provision of comparable experiences for users with disabilities. Travellers with visual impairments were involved in a collaborative process in all project phases, including problem identification, technical feasibility, proof of concept, design and development. Participatory design contributed to a thorough understanding of the user perspective and allowed the app to be optimised for the needs of travellers with visual impairments. Furthermore, co-design proved to be an effective method for fostering awareness and knowledge about digital accessibility at all organisational levels.

## Introduction

Beginning a journey, visiting your loved ones, or commuting daily to work: There are many reasons for boarding a train. People with visual impairments also share this daily travel routine. However, this group of travellers may face additional challenges on their journeys by public transport. In Switzerland, where the present case study was conducted, no immediate acoustic or tactile information is available to aid people when they board trains. Announcements in trains are usually made only a few minutes before departure, which can lead to stressful situations or even trips to the wrong destination. A similar dearth of information exists in train stations and on platforms. Currently, travellers with visual impairments have no direct means of accessing the information on departure boards listing the next available train connections. There are alternative means for obtaining this information; for instance, by querying the timetable provided in the mobile app of the Swiss Federal Railways. Nonetheless, such alternatives require extra effort, and the experience is hardly comparable to a quick glance at a departure board. As a consequence, this information gap may substantially limit a traveller’s autonomy and comfort.

Information systems installed in trains, train stations and on platforms have long life cycles, making it more difficult to address information gaps that were not previously considered. Digital technologies, such as smartphones or smartwatches, can provide an alternative means of access. Although these personal devices are widely available and have become pervasive in everyday life, they still do not guarantee a comparable experience [1]. Comparable experience here refers to digital information and services that are comparable in value, quality and efficiency for each user [9]. For instance, the ease of the aforementioned quick glance at the departure board could be replicated for travellers with disabilities by equipping apps with device features such as geolocation and screen reader support, thereby providing an efficient alternative means for them to interact with local information. Thus, designing comparable experiences for as many people as possible requires more than the mere transfer of identical content and functionalities to other technologies.

### Access to Experience

Power, Cairns and Barlet [7] describe three layers involved in achieving comparable experiences for users with disabilities. The authors refer to the work on digital accessibility that focuses on providing basic access to technologies as *First Wave Inclusion*. Basic access can be provided by offering alternative input modes (e.g., switch access that replaces interactions via touchscreen for users with limited dexterity in their hands) and by translating information into alternative output modalities (e.g., access by screen reader for users with visual impairments). While work on basic access remains vital for digital accessibility, recent work has broadened the perspective and moved away from the focus on mainly technical aspects [5, 8].

*Second Wave Inclusion* shifts the perspective towards enabling users with disabilities to achieve their goals [7]. This leads to a more usability-oriented understanding of digital accessibility, including traditional criteria such as effectiveness, efficiency and satisfaction in a specified context of use [5]. The opinion that digital accessibility and usability are related concepts is regularly discussed in research [6] and is widely accepted by accessibility experts [12]. Further, the understanding that this relationship benefits the overall quality of a product was shown to be a main motivation for considering digital accessibility [10].

Based on the analyses of access and enablement covered in the two previous layers, *Third Wave Inclusion* focuses on understanding the subjective experiences of users with disabilities in an interactive system [7]. This perspective goes beyond performance-related criteria and includes aspects related to user experience such as affect, trust or aesthetics [1]. However, in the digital accessibility field, this more holistic perspective is rarely adopted [4], and only a few tools and techniques have been developed to capture the subjective experience of users with disabilities [7]. A frequently cited approach is *participatory design*, in which users with disabilities actively define and shape the design of a product [4]. Participatory design contrasts with traditional user-centred design methods that involve users but leave their design decisions to be made by a project team of specialised professionals.

In the present case study, we detail the development of a mobile app, which was co-designed by travellers with visual impairments. The project was conducted in collaboration with the Swiss Federal Railways and pursued the goal of identifying and closing crucial information gaps that hinder travellers with disabilities during their journeys by public transport. Rather than giving specific advice on how to implement an accessible app, this report aims at providing insights into a participatory design process and also inspiring similar activities in other projects.

## Case Study

### Problem Identification

In a first phase, we asked travellers with disabilities to provide us with their experiences regarding any information gaps that they confronted during journeys by public transport. For this purpose, we contacted the Advisory Board for Barrier-free Travel of the Swiss Federal Railways, which represents travellers with visual, hearing and motor impairments. The board consisted of one person with a central scotoma since childhood (m, 45), two people who have been almost blind since childhood (f, 42; m, 59), one person with severe hearing loss since early childhood (f, 51), one person with age-related hearing loss (m, 77), one person with cerebral palsy since birth (m, 42), a low vision optician (m, 62), an acoustician (m, 80) and an expert in the field of barrier-free public transport (m, 71).

In two workshops, we mapped a complete user journey, ranging from planning, arriving at the station, finding the platform, boarding the train, travelling to the destination, and orienting oneself after arrival. For each part of the user journey, the representatives of the advisory board introduced potential information gaps and rated these according to their severity. Later, these insights were enriched with observations from first-hand experiences on an exemplary journey. For instance, the representatives with visual impairments demonstrated their lack of information when boarding a train by giving the non-disabled project members simulation glasses so that they could experience this issue personally.

### Technical Feasibility

Detailed problem descriptions derived from the user journeys were used as input for a *hackathon*. In a hackathon, teams of programmers and other specialists involved in software development collaborate intensively on a given project over a few days. One of the teams, including a blind programmer (m, 44), focused on information availability when boarding a train. With the development of an app using Bluetooth beacons and publicly available information from the Swiss public transport’s open-data platform^1^, the team was able to prove the app’s technical feasibility and its compatibility with assistive technologies. A few weeks later, another team extended the prototype in a second hackathon with regard to information availability in train stations and on platforms. By using geofencing based on GPS positioning, it was possible to provide a digital version of the departure boards which showed the next available train connections at the current position.

### Proof of Concept

We decided to further develop the ideas created in the technical feasibility phase for multiple reasons. First, the two aforementioned issues belonged to the most pressing information gaps for travellers with visual impairments. Second, the proposed solutions showed potential for being extended to travellers with other forms of disability; for example, by providing acoustic announcements in text form for people with hearing impairments. Third, another project involved installing Bluetooth beacons on a selection of train lines to test a different application, which allowed us to start our project immediately using existing infrastructure. A basic test app applying components built in the technical feasibility phase was distributed to a group of 34 interested travellers with visual impairments. The participants regularly travelled on specific train lines that were already equipped with Bluetooth beacons. They provided feedback via their communication channel of choice (e.g., via email, phone or voice messages). In addition, we conducted three exemplary journeys with a total of 10 travellers (6 women, 4 men; 5 blind, 5 with severe visual impairments) to discuss the app’s functionalities and design in a real context. During the proof of concept phase, we collaboratively created first drafts for the final product design. For instance, the test app featured the concept of the master-detail pattern, providing a short overview of the travel information with an option to see more content. Participants deemed this concept as impractical in the present context, since it requires browsing through an often changing list and an additional click to look for further information. In collaboration with the participants, a concept using tab navigation at the bottom end of the app and reserved areas for the most important information was outlined. These reserved areas have a fixed position on the screen and enable quick access and orientation using a screen reader.

To decide whether to continue the project, the proof of concept phase was closed with a questionnaire answered by a total of 14 participants (age *M* = 55.3, *SD* = 10.3, range 30–71; 4 women, 10 men; 7 blind, 4 with severe visual impairments, 3 with light visual impairments). Participants used the test app with various combinations of assistive technologies, including screen reader and voice control (*N* = 6), screen reader, voice control and inverted colours (3), screen reader only (3), and screen magnification (1). They rated the overall impression of the test app positive (*M* = 4.23; *SD* = 1.1; 1 = *worst rating*, 5 = *best rating*). To gain further support for the project, we decided to use the test app to spread awareness of digital accessibility issues within the organisation. In 3 workshops, a total of 60 employees of the Swiss Federal Railways were invited to personally experience the addressed information gaps. Travellers with visual impairments were present during these workshops and shared their experiences in dealing with these issues.

### Design

Based on the insights generated in the previous phases, we compiled a final conceptual design. The app was named SBB Inclusive (i.e., SBB stands for Schweizer Bundesbahnen, Swiss Federal Railways). Next, we asked four blind users (age *M* = 46.7, *SD* = 14.4, range 30–65; 1 woman, 3 men) to participate in a usability test, in which they solved typical tasks with an early prototype [3]. Often, such tests are carried out with pen and paper or wireframes, which cannot be accessed directly by users with visual impairments. A simple web prototype built using HTML and CSS proved to be an effective workaround. This allowed us to test the navigation structure, the order of the displayed elements, and the richness of information directly using a screen reader. Participants had the choice between using either a test device or their own personal device. This allowed them to participate in the test while using their own familiar settings, such as their personal screen reader speech rate. To refine the concept, we discussed findings with the participants immediately after each test session and collaboratively outlined design improvements.

**Fig. 1.**
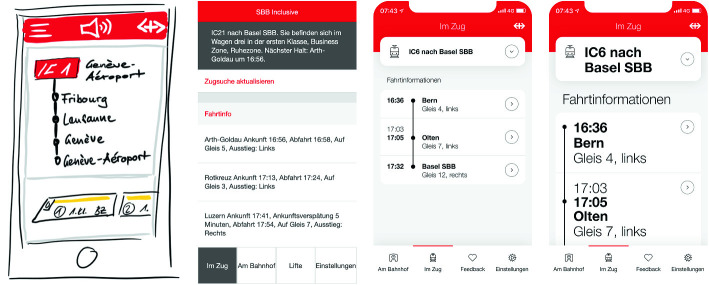
Evolution of the app during the design phase. From left to right: first scribbles, HTML prototype, final design and final design with increased text size.

Finally, we created a visual design for the app, taking the accessibility features of the operating systems into account. For instance, a specific screen layout was designed for large text settings, which allows for text resizing without loss of content or functionality. The evolution of the app during the design phase is presented in Fig. 1.

### Development

An app version for iOS using SwiftUI and a version for Android using Flutter were created from scratch. We deemed both technologies as being optimally suited for building the intended features. Travellers with visual impairments who participated in the proof of concept phase were invited to upgrade their apps to the new app and were asked to give their feedback on the ongoing development using a built-in contact form. At the date of this publication, the new app was just made available for testing. Therefore, little feedback has been received so far: however, most of it expresses a positive first impression of the final version of the app. The public release of SBB Inclusive is planned for fall 2020.

### Future Development

With regard to future development, we plan to focus on additional information gaps that were revealed in the problem identification phase. Current ideas include developing features to display acoustic announcements in text form to travellers with hearing impairments, to monitor the status of elevators in train stations for travellers with physical impairments, and to provide information in reduced language complexity for travellers with cognitive and learning disabilities.

## Discussion

Travellers with visual impairments participated in all phases of the present case study. The co-design process allowed us to obtain a thorough understanding of information gaps during journeys by public transport from a user’s perspective. Especially, the workshops with representatives from the advisory board in the problem identification phase and the exemplary journeys in the proof of concept phase proved to be helpful for this purpose. These occasions also created a space for collaboratively drafting ideas, which led to the conceptual design used in the final product. In the present case study, participatory design allowed us to attain a level of quality which would arguably not have been achieved with traditional user-centred design methods.

Further, the shared understanding provided a solid basis for the development phase of the app, which required continuous design decisions that had to be in line with user needs. For this mainly technical phase, it would have been a major advantage to have a person with visual impairment as a fixed member of the development team [4]. This was partly the case during the technical feasibility phase and proved vitally important for the iterative testing of solutions, for integrating resources from first-hand experiences into the product, and for receiving hints on how similar functionalities are solved in other apps. Future projects should extend participation to all project phases and staff a more diverse development team. At the same time, close involvement of a broad user group should be maintained. Such a setup allows for a balance between the expertise of a project team and the perspectives of unbiased users.

Another insight from the present case study was the importance of the impact that participatory design has on stakeholders within the organisation. Recommendations to involve users with disabilities in the development process in order to foster awareness at all organisational levels were put into practice effectively [10]. In particular, the proof of concept phase with its workshops which allowed participants to personally experience the inconvenience caused by the information gaps proved to be an effective tool for promoting knowledge about digital accessibility within the organisation. Internal stakeholders who were initially somewhat indifferent to information gaps for travellers with disabilities soon saw the importance of these issues while collaborating with travellers with visual impairments. The involvement also contributed to a reduction in misconceptions regarding digital accessibility; for instance, the prevalent belief among stakeholders that aesthetics and technologically advanced products would be compromised by introducing accessible solutions [2]. An extension of the participatory design process to other groups of users with disabilities would benefit this promotional effect. Since the scope of digital accessibility often centres around users with visual impairments [11], such a step could broaden an organisation’s awareness for various perspectives and motivate it to invest in providing comparable experiences for all user groups. Another benefit of involving internal stakeholders closely was the opportunity to exploit synergies with other projects. The possibility to reuse an existing technical infrastructure was crucial to obtaining the technical solution described in the present case study, as this allowed the project to start immediately and shortened the implementation time substantially. Perhaps, there will be further synergies in other contexts that can be used in a creative way to support digital accessibility.

## Conclusion

In the present case study, a mobile app was co-designed by travellers with visual impairments to create a user experience that is comparable in value, quality and efficiency to that of non-disabled travellers. Participatory design contributed to a thorough understanding of the user perspective and allowed us to optimise the app to the needs of travellers with visual impairments. By extending the use of participatory design to all development phases and by staffing projects with a more diverse team, these observed benefits could be further employed in future projects. Interactions between travellers with visual impairments and the stakeholders within the organisation helped to spread accessibility awareness and knowledge at all organisational levels and triggered synergies with other projects. Future work should broaden the spectrum of disabilities considered to include as many people in as many situations as possible. We hope that our research will encourage project teams to benefit from a wide range of user perspectives in order to improve their work.

## Footnotes

1 https://opentransportdata.swiss/en.
